# Measurement dataset of experimental in-body optical wireless communication test-bed for research purposes

**DOI:** 10.1016/j.dib.2025.111765

**Published:** 2025-06-09

**Authors:** Syifaul Fuada, Mariella Särestöniemi, Marcos Katz

**Affiliations:** a Centre for Wireless Communications, Faculty of Information Technology and Electrical Engineering, University of Oulu, Oulu 90570, Finland; b Research Unit of Health Sciences and Technology, Faculty of Medicine, University of Oulu, Oulu 90570, Finland; c InfoTech Oulu, University of Oulu, Oulu 90570, Finland

**Keywords:** Near infra-red (NIR), Pork meat sample, Optical wireless communications (OWC), In-body communication, Test-bed, Light

## Abstract

Ultra-wideband (UWB) and narrowband (NB) technologies have been widely used for in-body communication systems. In recent years, there has been a growing interest among researchers in optical wireless communication (OWC) as an alternative technology for in-body communication; this trend has emerged as a response to the limitations and challenges found in UWB and NB communication technologies. Conducting an in-body OWC study using an *ex-vivo* approach should consider several key steps. Initially, a standardized test-bed must be prepared; this involves developing it step-by-step with commercial off-the-shelf components (COTS), followed by a thorough characterization/assesment of its performance. This data-in-brief paper provides a set of measurement data obtained from a developed test-bed for in-body OWC research based on phantom and *ex-vivo* samples. The methodology used for data collection and the significance of the measurements are explained. The test-bed employed two receiver ($R_{x}$) devices representing an in-body device, namely 1) a photodetector module and 2) an optical sensor connected to an optical power meter console, where both devices are created by the same company (i.e., Thorlabs). The data includes the results of three measurement scenarios, namely 1) free-space channel (baseline case), 2) tissue-mimicking optical phantoms, and 3) biological tissue channels based on *ex-vivo* samples of fresh pork meat of different compositions. The uniqueness of this test-bed lies in its use of a photodetector module to serve as an optical power meter and then comparing the result to the optical power meter readings under three measurement scenarios (i.e., free-space, phantoms, and biological tissue samples). In addition to its primary role of converting information signals in the optical domain into the electrical domain, the photodetector module can be used indirectly to measure optical power by using the equations outlined in the datasheet, extracting the output voltage ($V_{out}$) to determine the relative optical power. The dataset presents the impact of varying incident power of near infra-red (NIR) LED, achieved through adjustments in LED current using the LED driver module, on the received optical power measured by an optical power meter and photodetector module in a separate measurement. The influence of the photodetector’s gain setting on the received optical power read by a photodetector module is also investigated. From the top-level perspective, the developed test-bed confirms its feasibility in demonstrating in-body OWC systems. From the specific point-of-view, data obtained in this paper suggests two main findings: **first**, changing the photodetector’s gain can increase the $V_{out}$, but it does not affect the measured optical power based on the calculation. Gain adjustment can serve to increase the scale of $V_{out}$ reading. **Second**, the received optical power read by the photodetector module in any gain setting is closely matched with the optical power meter reading set to approximately –4.30 dB. In this sense, multiplication should be considered to align the results of optical power readings between the photodetector module and the optical power meter when using them in the experiment at the free-space and *ex-vivo* settings, which is around 2.7×. Future use of the provided data is intended for researchers in the biomedical engineering field, particularly those focusing on in-body OWC and dealing with *ex-vivo* experiments. This dataset paper can facilitate the procedure of developing and testing an in-body OWC system on a laboratory scale using the standardized test-bed, providing inspiration to researchers in this area who wish to use a similar setting with comparable instruments.

Specifications Table

**Table:** 

Subject	Physical sciences: Optics
Specific subject area	A data-in-brief article is related to optics (i.e., optical wireless communication topic) under the subject of physical sciences. The paper presents the characterization and measurement of a test-bed dedicated to research on in-body optical wireless communication (OWC).
Type of data	Raw (.xlsx file)
Data collection	The test-bed demonstrator of in-body OWC consists of transmitter ($T_{x}$) and $R_{x}$ front-ends based on Thorlabs equipment. The $T_{x}$ includes a 375 mW near infra-red (NIR) LED with λ = 810 nm and an LED driver module (DC2200) for controlling the LED’s current. The $R_{x}$ device comprises two types of detectors: a photodetector module (PDA36A-EC, λ = 400 to 1000 nm) and an optical power meter module (PM100D) integrated with an optical sensor (S121C). The dataset considers the influence of different levels of LED current control (50, 100, 150, 200, 250, 300, 350, 400, 450, and 500 mA) on the photodetector module (gain = 0 dB and 10 dB) and the optical power meter (attenuation = 0 dB and –4.30 dB) readings. The dataset includes three scenarios: 1) free-space measurement, 2) through phantom and 3) across biological tissue channels. We also measured the incident optical power of the NIR LED under different levels of LED current control. On a free-space setting, we measured the received power at 5, 7.5, 10, 12.5, and 15 cm of optical distance. On phantoms, we measured the received power on 1 cm and 4 cm of thicknesses. We measured the received power on biological tissue using a 3.5 cm thick sample of fresh pork meat consisting of different layers, i.e., fat, muscle, and bone. The received optical power for these three scenarios (i.e., free-space, phantom, and biological tissue) was measured using a photodetector module by converting its $V_{out}$ read by an oscilloscope (54845A HP Infinium 1.5 GHz 8 GSa/s). Further, the optical power results extracted from the photodetector module were compared with an optical power meter, which was set to 0 dB (ideal setting) and then –4.30 dB (adjusting level to match with reading results with a photodetector module). The measurements were conducted in a very low-intensity setting to avoid light interference from other light sources within the laboratory, approximately 0 lux.
Data source location	Experimental design and measurement were performed at the Wireless Medical Communications (WiMec) Research Group, Centre for Wireless Communications, Faculty of ITEE, University of Oulu (TS253, VLC Laboratory, Pentti Kaiteran katu 1, 90570 Oulu, Finland). The phantoms used for the experiment were based on recipes available from [1,2]. The fresh pork meat samples used for the *ex-vivo* experiment were purchased from a local market in the city of Oulu: Liha & Kalakauppa (Ilmarinkatu 16 FI-90400 Oulu, Finland). Site: https://lihajakalakauppa.fi/ .
Data accessibility	Repository name: Test-bed preparation for in-body optical wireless communication studies [3] Data identification number: 10.17632/zhm4kn6sfn.1/3 Direct URL to data: Mendeley repository (https://data.mendeley.com/datasets/zhm4kn6sfn/3)

## 1 Value of the Data

**Fig. 1:**
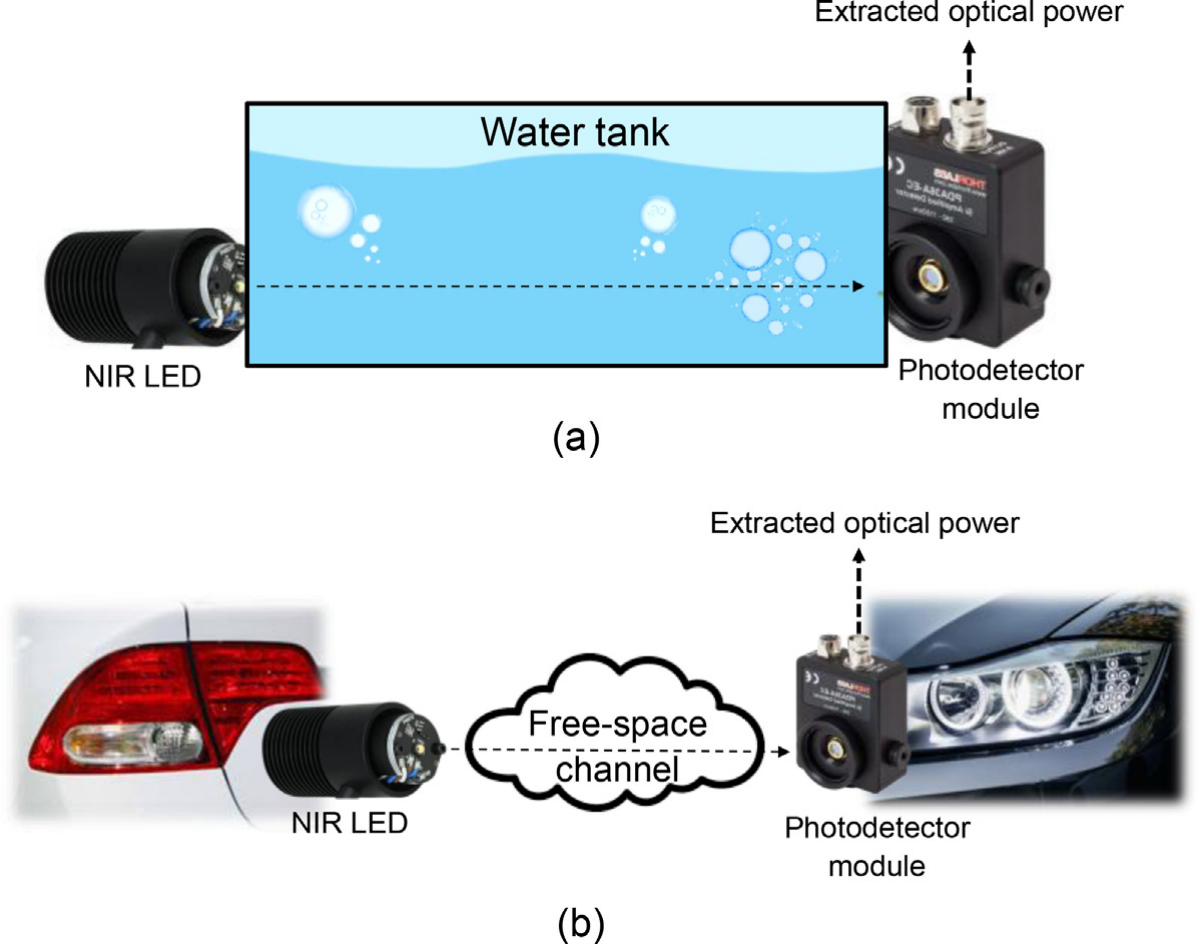
Example of the most possible use cases: (a) underwater communication based on OWC; (b) OWC system in outdoor settings, e.g., car-to-car communications.
• The existence of this dataset may enable further study on different disciplines, for instance, the security level enhancement of in-body communication; an example of the implementation can be found in [4], which uses data from optical power meter readings extracted from the photodetector's output voltage ($V_{out}$), to generate a new dataset for securing wireless body area network (WBAN) systems.
• Researchers working in the area of energy harvesting (EH) or optical wireless power transfer (OWPT) through free-space settings (e.g., [5]) using NIR spectrum and photovoltaic cells (PV) can benefit from this data. Our test-bed is well-suited for free-space settings (especially for optical distances around 5 – 15 cm), making it applicable to the OWPT research field.
  Researchers working on OWPT using NIR light through biological tissue can also utilize this dataset paper [6], allowing them to develop a test-bed on a free-tissue sample [7]. The inefficiency of PV cells in capturing NIR light would be a challenge to conducting OWPT research through biological tissue. Given that the reading process to fully charge the battery may take a few hours due to the mismatch between the wide spectral response of PV cells and the narrow spectral output of the used NIR light, readings of harvested energy on a free-space can be conducted without any issue. However, when working with biological tissues, problems will arise as the quality of the sample may degrade over time during power transmission. One possible solution to overcome this is to consider a free-tissue sample experiment (conducted in a free-space channel). This can be achieved by following the following steps: First, place the meat sample between the LED and the optical power meter. Second, adjust the LED power and record the initial received power on the $R_{x}$ side. Third, remove the meat sample. Fourth, readjust the incident power of the LED until the received power, as measured by the optical power meter, closely matches the results obtained when a biological sample is present (the detailed discussion is elaborated in a different dataset paper publication [7]). Using our dataset, other researchers can bypass this challenging setup by utilizing the test-bed proposed in this data-in-brief paper. In this context, the received optical power can be measured using either an optical power meter or a photodetector module (serving as an alternative).

• OWC systems have fundamental advantages that can be employed as the baseline of in-body communication for future implant technologies. This dataset can serve as a reference for other researchers looking to build a standardized test-bed for research purposes on in-body OWC systems inspired by UWB technology. Additionally, the data is described as remarkable supplementary information for the original company regarding the characteristics of their products.
• This unique dataset compares, for the first time, a commercial optical power meter and photodetector module to build an in-body OWC system test-bed in terms of optical power (in Watt units). The data also suggests that the photodetector module, which most researchers employ only for demonstrating OWC performance or proofing the concept of the optical system, can also be utilized as a device for measuring relative optical power by a particular methodology and requirement, especially applications under NIR light. Optical power meters are one of the equipment that play a crucial role in measuring the performance of optical systems, particularly in assessing received absolute optical power across various ecosystems. This dataset specifically focuses on indoor free-space, phantom, and biological tissue settings under NIR light. We anticipate that the methodology provided to create this dataset can be applied in research contexts beyond the in-body OWC use case. For instance, we assume that it may be utilized for measuring optical power in underwater scenarios (e.g., underwater OWC) or outdoor applications (e.g., vehicle-to-vehicle OWC), as illustrated in Fig. 1.

## 2 Background

Over the last few decades, in-body device (e.g., implantable medical devices “IMDs”, injectable devices, ingestible devices, etc.) technology has rapidly been developed with the advances of multidiscipline emerging scientific and engineering technologies [8]. In-body communication enables communication in two directions: in-body to the external access point and vice versa. The in-body device is a key part of the medical equipment that monitors physiological data inside the human body and transmits it wirelessly to an external access point. In this case, in-body communication serves as a biotelemetry application (remote monitoring) where an in-body device works by transmitting the biosensing signal (e.g., health records or other medical data) through the tissue channels. On the other hand, data (e.g., configuration settings, user profile modifications, and other related commands) can be transmitted from the external device to the in-body device, which is capable of being controlled externally. This functionality helps medical professionals or doctors to manage these tasks wirelessly. In this sense, in-body communication serves as a remote adjustment for the in-body device operation.

UWB technology has been mainly used in the in-body communication system since it enables low-power, low-cost, safe, and reliable communication between the in-body and the on-body device. Despite the several advantages of UWB systems, their limited propagation capabilities result in very short communication ranges. Narrow Band (NB) radio communications, e.g., *industrial, scientific, and medical* (ISM) band 2.45 GHZ, can be used for in-body communication systems. However, NB-based technology is more likely to be exposed to cyber-attacks due to radio frequency (RF) communications security issues. Optical wireless communications (OWC) can undoubtedly overcome the drawbacks of NB because of its diverse advantages for in-body devices, e.g., increased security, noninterference, harmlessness for the human body, safety, and privacy. On the other hand, the reason behind utilizing optical instead of UWB is to eliminate the drawbacks associated with UWB, such as lack of safety (e.g., specific absorption rate). Examples of OWC for in-body communication utilizing the visible light spectrum can be found in [9,10]. The light penetration depth across the biological tissues is highly affected by the absorption, reflection, and scattering issues. When interacting with the visible light spectrum, biological tissues absorb and scatter it significantly. Thus, visible light, as in [9,10], has a low penetration depth in biological tissues, which is limited to a few millimetres, making it challenging for deeply in-body devices. However, the use of NIR wavelengths (around 700–1100 nm) can mitigate these issues (i.e., less absorption, reflection, and scattering effects); thus, the optical link range and efficiency for data exchange can be enhanced as it can penetrate biological tissues more effectively than visible light. To this end, NIR LED is more favorable for this application.

**Fig. 2:**
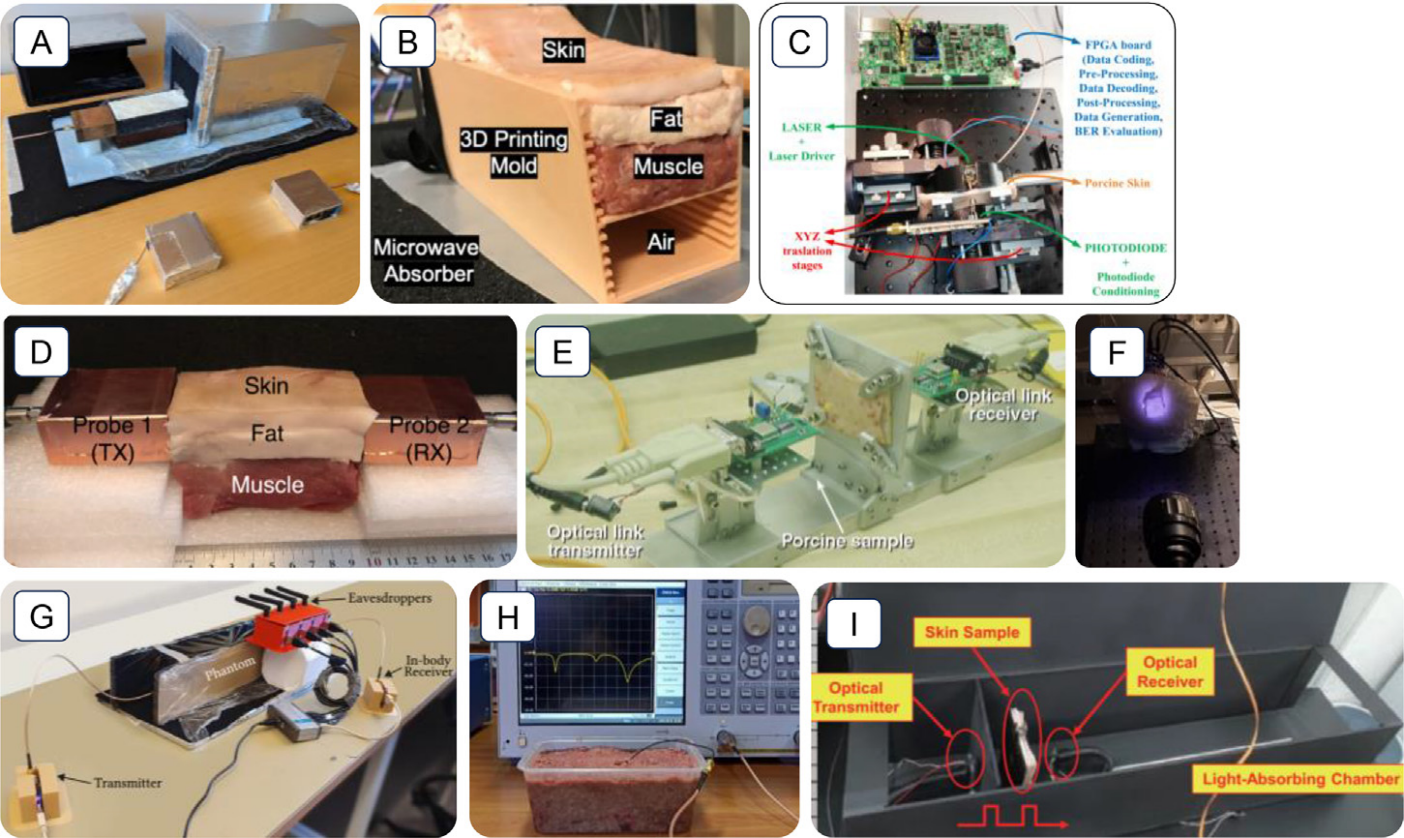
Example of test-bed in in-body communication research based on OWC and RF technologies: (a) Ref [13]; (b) Ref [14]; (c) Ref [15]; (d) Ref [16,17]; (e) Ref [18]; (f) Ref [19], (g) [20]; (h) Ref [21]; (i), and Ref [22].

Experimental test-beds are models to acquire insights into specific elements of the system. Constructing test-beds will aid in assessing interconnected solutions to system issues and enable design choices grounded in theoretical or empirical research. The main goal of these test-bed implementations is to conduct various experiments, considering a careful balance between controllability in laboratory settings and resemblance to actual conditions [11]. The in-body OWC test-bed provides a more realistic and practical representation of real-world clinical applications compared to simulation-based environments. In the literature, numerous innovative test-bed designs have been developed and demonstrated by scientists for in-body communication research, as shown in Fig. 2. Currently, there are no golden standards for test-beds looks like. Therefore, scientists can create it on their own. They are typically customized based on available equipment or can be called commercial off-the-shelf components (COTS). The test-bed built by COTS is more favorable than the test-bed based on the application-specific integrated circuits (ASICs) approach as it is envisioned to have a faster deployment (reduce the chip design and development cycles) and a cost-efficient alternative. On the other hand, it allows rapid prototyping and reshaping of in-body devices [12].

In the context of in-body OWC test-beds based on COTS, they are dedicated to evaluating specific aspects of wireless communication across biological tissues, such as signal attenuation, light propagation characteristics, and data transmission performance. Standard test-beds for in-body communication research at least involve a $T_{x}$ antenna, a sample as a medium (e.g., *ex-vivo* pork meat or phantom), an $R_{x}$ antenna and its measuring tools. Typically, the scientist develops their own holders or containers to adequately store a sample, as shown in Fig. 2.

It is expected that the use of a standardized test-bed would enhance the reproducibility of experiments on in-body communication. However, considering in-body OWC test-bed development, several challenges are faced to meet the desired specifications, including hardware preparation and characterization. We found that the dataset for test-bed characterization is not yet available in the literature. It is encouraged that these data should be accessible, as they are of utmost importance given the increasing demand for in-body communication utilizing the optical spectrum. Motivated by those, we provide measurement data of test-bed preparation dedicated to in-body OWC research.

This dataset paper contains four sections. The first is the value of the data, which explains the utility of the data; we highlight several key points regarding the significance and potential applications of the dataset for the research community. The second is the background of the dataset paper. Third is data description; we provide a detailed breakdown of the datasets related to the test-bed preparation for in-body OWC research, including 1) the type of data, structure and content of the dataset files, 2) specific components of the test-bed demonstrator used in the experiments, 3) the parameters measured, and 4) experimental conditions (i.e., experimental setup involving LED current control, photodetector, gain settings, and measurement of received optical power in different scenarios which are free-space measurements, through phantom and biological tissues). Fourth is the experimental design, materials, and methods; we describe the protocol and method for generating the data. In this section, we detail the instruments and equipment used, the experimental setup, the procedures for conducting measurements, and the calculations performed to derive the data.

**Contributions:** To the best of our knowledge, the data presented in this dataset paper is novel and specifically related to the development and characterization of the test-bed for in-body OWC research. The dataset enables comparisons between a commercial optical power meter and a photodetector module for building in-body OWC system test-beds, providing insights into optical power measurement methodologies. The dataset can be used for future research in in-body OWC and has the potential for standardizing test-beds.

## 3 Data Description

This article provides datasets prepared for test-bed purposes prior to conducting experiments on in-body OWC. The datasets are enclosed in three Excel files with the extension *.xlsx*, which can be accessed in the repository. Below is a detailed description of each file. The section also provides details on data accessibility, including the repository name, data identification number, and a direct URL to access the dataset on the repository. In the repository, we provided a “read me” file to make it easy for users, highlighting how to use our dataset. The “read me” file also serves as a guide for those new to the equipment used, experimental setup, and data analysis methods.

### 3.1 Raw data - optical test-bed in-body communications (free-space)

**Fig. 3:**
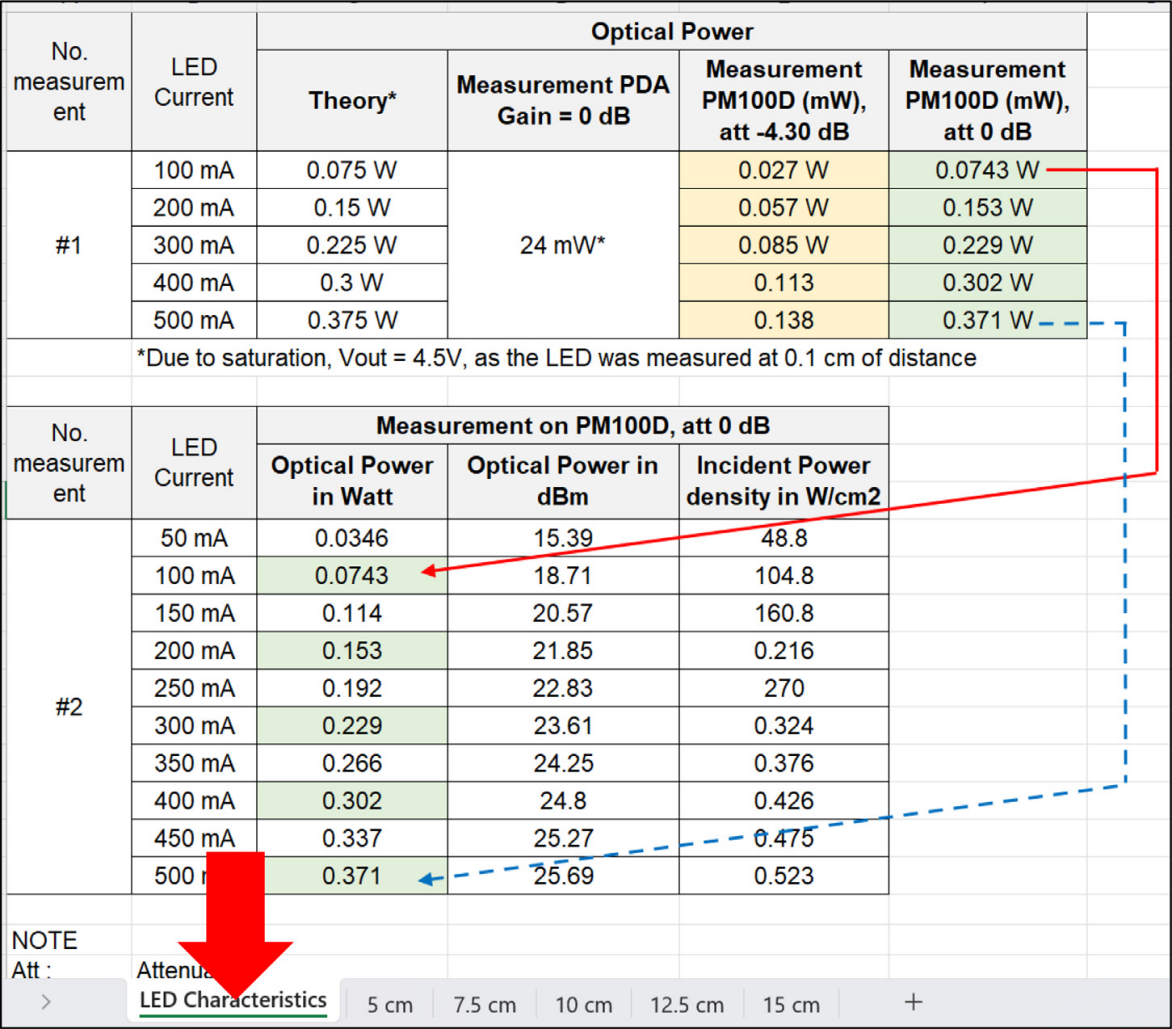
Description of the raw data of sheet 1.

The file can be accessed at [3], or simply click the following link to direct access: https://data.mendeley.com/datasets/zhm4kn6sfn/3. After downloading successfully, open the *.xls* file and look at the file. The file consists of several sheets. The first sheet provides data on measuring the incident optical power characteristics of the NIR LED used in the test-bed. This data includes two experiments labeled “Experiment *#1*” and “Experiment *#2*”, as shown in Fig. 3. Experiment *#1* provides information on the current supplied to the NIR LED through the LED driver module (i.e., 50, 100, 200, 300, 400, and 500 mA). The data is derived from the datasheet of the NIR LED (theory) [23] and incorporates measurements from the optical power meter, with attenuation = 0 dB (PM100D) and the photodetector module (PDA36A-EC) using gain = 0 dB, expressed in Watt units. Theory refers to the calculation by seeing the datasheet on the correlation between the incident power and driving current. The maximum incident power is 0.375 W when driven at the maximum point, i.e., 500 mA (100% of capacity) [23]. If the NIR LED is driven at 100 mA (approximately 20%), it would be 20% of 0.375 W, which is 0.075 W. Experiment *#1* shows that measurement using an optical power meter at an attenuation = 0 dB is quite similar to the theory, for instance, 0.375 W in theory and 0.371 W in actual measurement. We compared the results using attenuation = –4.30 dB and a photodetector module at a gain = 0 dB. As expected, the photodetector cannot measure the incident power since it is saturated, and attenuating the received signal directly is not possible in the photodetector module.

Experiment number *#2* solely presents measurements from the optical power meter at an attenuation = 0 dB, which are converted to optical power (dBm units) and incident power density (mW/cm^2^ units). Experiment *#2* employs LED driver settings at 50, 100, 150, 200, 250, 300, 350, 400, 450, and 500 mA. For instance, the corresponding optical power in Watt, in dBm, and power density of NIR LED when driving at 100 mA are 0.743 mW, 18.71 dBm, and 104.8 mW/cm^2^, respectively.

**Fig. 4:**
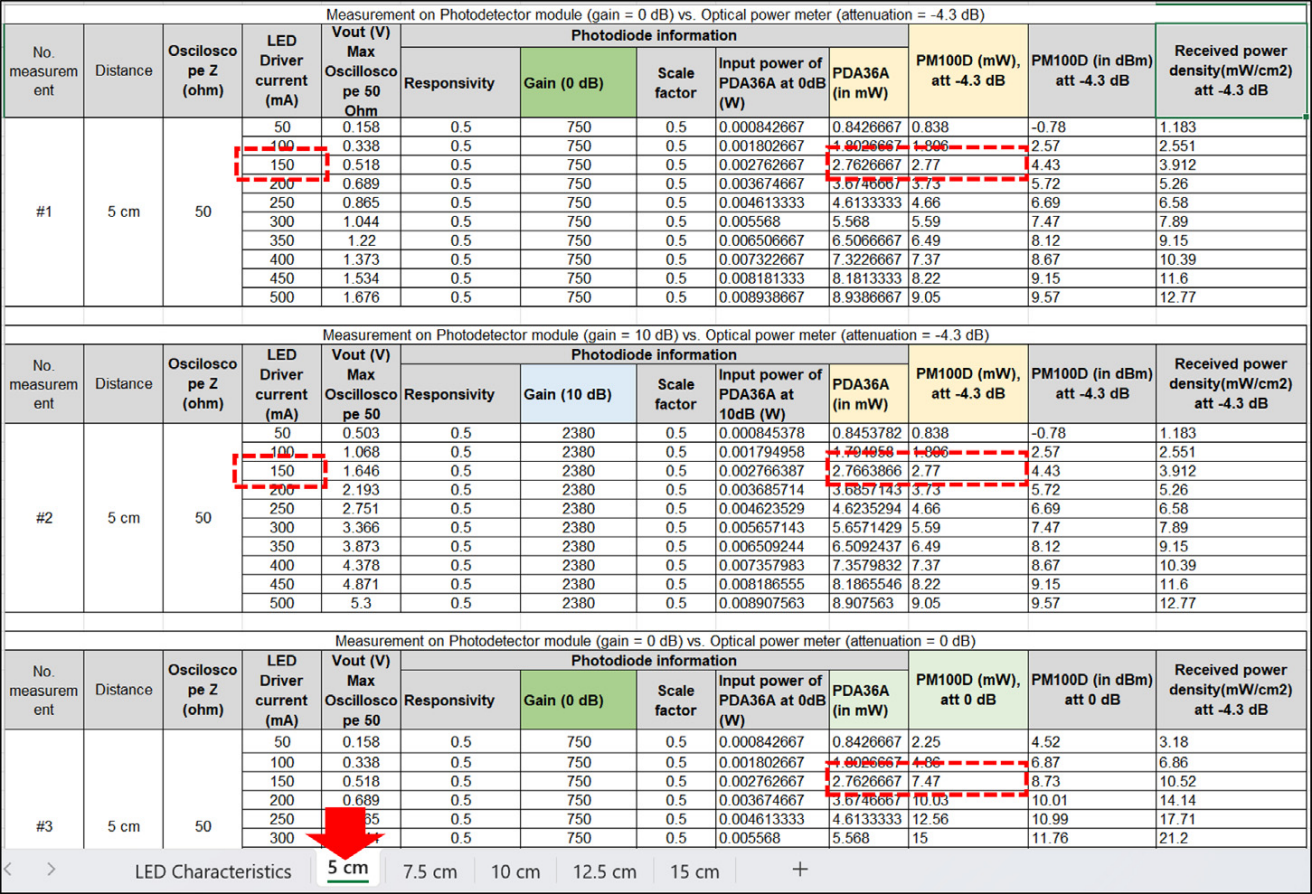
Description of the raw data of sheet 2.

The second sheet provides measurement data on a 5 cm free-space. It comprises nine columns, including the measurement number (*#1, #2*, or *#3*), the distance of the channel (5 cm), the impedance of the oscilloscope utilized in the measurement (50Ω), the current applied to the LED driver (from 50 to 500 mA), the $V_{out}$ from the photodetector module displayed from the oscilloscope, photodetector information, and the optical power meter attenuation setting (using attenuation 0 dB or –4.3 dB). The value of the photodetector is based on the gain value set on the knob (either 0 dB or 10 dB), taken from the datasheet [24]. Fig. 4 shows the appearance of sheet 2. Experiment *#1* provides a comparison between photodetector reading at gain = 0 dB and optical power meter at attenuation = –4.3 dB. We use colour in the measurement table to indicate the same information; for instance, gain at 0 dB = 750 V/A is the same value in experiments *#1* and *#3*, etc. We inserted a formula in the “input power of PDA36A” based on the equation available in the photodetector’s datasheet [24], in watt unit and subsequently in mW unit by multiplying the calculation result with 1000. The measurement results presented in the dataset using an optical power meter include mW, dBm, and mW/cm^2^ units. For instance, as demonstrated in experiment *#1*, the oscilloscope displayed $V_{out}$ = 0.518 $V_{pp}\;$when the NIR LED emitted a stable illumination, driving at 150 mA current. The corresponding received optical power from the photodetector module (i.e., PDA36A-EC) reading result is 2.76 mW, which was quite similar to the received optical power displayed in the PM100D, which was 2.77 mW. Experiment *#2* provides a comparison between the photodetector reading at gain = 10 dB and the optical power meter at attenuation –4.3 dB. As shown in experiment *#2*, the received optical power obtained from the photodetector reading result at emitted NIR LED driving by 100 mA current is 2.76 mW (with the value of $V_{out}$ = 1.646 $V_{pp}$). The measurement result confirms that *changing the photodetector’s gain (e.g., 0 dB, 10 dB, etc.) does not affect the measured optical power*; gain adjustment only serves to increase the reading scale of the DC signal from the photodetector output. Experiment #3 provides a comparison between the photodetector reading at gain = 0 dB and the optical power meter at attenuation = 0 dB. The received optical power displayed in the PM100D using attenuation = 0 dB is 7.47 mW, which is almost 2.7× the photodetector reading.

The data on the third, fourth, fifth, and sixth sheets are based on measurements taken at 7.5, 10, 12.5, and 15 cm in free-space, respectively. The data presentation format on these sheets is the same as the second sheet. The data is consistent in that adjusting the gain of the photodetector module (0 dB or 10 dB either) does not impact the measured optical power in free-space channel (i.e., 5, 7.5, 10, 12.5, and 15 cm). However, when the photodetector module is employed to measure optical power across the phantom and biological tissue channels, consideration should be taken into account to align with the received optical power of the optical power meter.

### 3.2 Raw data- optical test-bed in-body communications (*phantom*)

This data is also available for download on the Mendeley repository, as mentioned before. The data file exhibits a similar presentation to the file titled “Raw data - optical test-bed in-body communications (free-space)” found on sheets 2 until 6. In this data file, results for phantom 1 (4 cm thickness) and phantom 2 (1 cm thickness) are presented in sheets 1 and 2, respectively. The data demonstrates that the optical power reading obtained from the optical power meter PM100D through the phantom is equivalent to 2.7× that of the photodetector module (i.e., PDA36A-EC). A $V_{out}$ reading of 0 $V_{pp}$ (as presented in Phantom 1) signifies that the signal amplitude displayed in the oscilloscope is too low and, therefore, being interpreted as zero in this dataset. As shown in the file, the optical power meter PM100D offers superior accuracy as it can detect low NIR light penetrating across the phantom and is better suited for precise optical power measurements. PDA36A-EC is one of the photodetector modules, which is a useful and competitive tool for detecting light signals, particularly in applications that require measurement of relative optical power within a specific wavelength range. Its performance is especially advantageous in scenarios where maintaining measurement consistency across a defined wavelength band is critical. In this dataset paper, we utilized a wavelength of 810 nm. We applied it within a phantom, demonstrating the ability to deliver consistent and reliable results in measuring the optical power.

### 3.3 Raw data- optical test-bed in-body communications (*ex-vivo* pork meat sample)

The data can be found at the same link in the Mendeley repository, as aforementioned. In this data file, results for *ex-vivo* pork meat samples containing different tissue layers (i.e., fat, muscle, and bone) are presented. The propagation channel for this data is 3.5 cm, representing the sample thickness. The oscilloscope's reading results are too small, i.e., in the mV order; therefore, we provide an additional row to convert them into volts. The extracted optical power is in the µW order.

Table 1 provides a summary of the key contents of each file, including the types of measurements included, corresponding experimental conditions, and specific variables recorded in each sheet. This table enables readers to quickly grasp the dataset's organization before delving into the details. Furthermore, Table 1 maps the measurement tools and experimental settings used in three different measurement tests: free-space, *ex-vivo* porcine sample, and optical phantom.

**Table 1:** Key contents of each file available in the repository.

Filename	No. of sheets	No. of measurements	Optical distance	Corresponding measurement conditions			Types of measurement included	Specific variables recorded
				Settings PDA36AEC	PM100D	Overall settings		
Raw data - Optical test-bed In-body communications (Free-space).xls	6	*#1*	Free-space at 5, 7.5, 10, 12.5, and 15 cm.	Gain 0 dB	Attenuation 4.30 dB	• Dark condition • LED driver settings: 50, 100, 150, 200, 250, 300, 350, 400, 450, and 500 mA • Oscilloscope settings: 50 Ω of input impedance • PM100D settings: 9500 nm input aperture, high bandwidth (BW Hi), and 810 nm wavelength reading.	• Absolute received optical power measured by PM100D • The relative received optical power measured by PDA36A-EC and the reading results from the oscilloscope (in volts) are converted into received optical power (in watts) using equation (2). Several parameters for voltage and watt extraction in (2) are responsivity, gain, and scale factor.	Received optical power (in mW, dBm) and power density (in mW/cm^2^) using PM100D $V_{out}$ (in volts) of PDA36A-EC measured by the oscilloscope
		*#2*		Gain 10 dB	Attenuation 4.30 dB			
		*#3*		Gain 0 dB	Attenuation 0 dB			
Raw data - Optical test-bed In-body communications (ex-vivo pork meat sample).xls	1	Sample 1	3.5 cm	Gain 0 dB	Attenuation 4.30 dB			
				Gain 10 dB	Attenuation 4.30 dB			
				Gain 0 dB	Attenuation 0 dB			
Raw data - Optical test-bed In-body communications (Phantoms).xls	2	Phantom 1	4 cm	Gain 0 dB	Attenuation 4.30 dB			
				Gain 10 dB	Attenuation 4.30 dB			
				Gain 0 dB	Attenuation 0 dB			
		Phantom 2	1 cm	Gain 0 dB	Attenuation 4.30 dB			
				Gain 10 dB	Attenuation 4.30 dB			
				Gain 0 dB	Attenuation 0 dB			

## 4 Experimental Design, Materials and Methods

This section provides a detailed description of the methodology used in data collection, including step-by-step procedures summarizing the key steps and considerations for replicating the experiments to improve reproducibility and reusability, equipment setup, and measurement protocols, with the reason that it will help readers understand how the data was generated and ensure reproducibility. We also include details on calibration procedures and data validation techniques to ensure the reliability and accuracy of the dataset. There are four steps in the data collection procedure: pre-preparation, preparation and safety concerns, measurement, and generated dataset. A visual look of the systematic description of the protocol used to create the dataset is presented in the Appendix (Fig. 20).

### 4.1 Step 1: pre-preparation

#### 4.1.1 Component selection

Selecting the proper components for developing a standardized test-bed is crucial, and it should be the first concern. NIR light was chosen to illuminate the biological tissue as it has better propagation properties across tissues than other wavelengths [25], specifically between 800 and 900 nm [26]. Our setup employed mostly COTS-based, which were produced by Thorlabs (https://www.thorlabs.com/navigation.cfm?guide_id=1). Thorlabs is one of the companies that produce various optical equipment that is widely employed by scientists for numerous forms of optical studies (including the OWC field) due to its reliability, precision, and suitability for optical applications. For this reason, we implemented the experimental system to form a standardized in-body OWC test-bed.

The essential devices comprise the single-beam mounted NIR LED (M810L3), LED driver module (DC2200), photodetector module (PDA36A-EC), optical sensor (S121C), optical power meter (PM100D), and oscilloscope (54845A HP Infinium 1.5 GHz 8 GSa/s). Certified protective eyewear specific to the wavelength being used should be considered during the experiment, as working with NIR light sources requires careful attention to prevent any accidental eye exposure. Table 2 shows the functions and original source of each equipment; some of them are still active, and the rest are inactive (e.g., obsolete). Despite the company stopping to produce the particular component, the optical power meter and optical sensor used in this study have valid calibration dates. The details of calibration issues are elaborated on in a subsequent section.

**Table 2:** Information on the in-body OWC test-bed.

Parameters	Function	Reference
NIR LED	Serving as an optical antenna of the system, it emits NIR light in a specific wavelength	[23]
LED driver	To control stable LED currents precisely	[27]
Photodetector module	Serving as an $R_{x}$ device	[24]
Optical sensor	Serving as an $R_{x}$ device	[28]
Optical power meter	Serving as a console to set the received optical power reading as well as displaying the received optical power; this device is paired with an optical sensor	[29]
Oscilloscope	To display the DC signal from the photodetector module output (i.e., $V_{out}$); this device is paired with a photodetector module	[30]

**Fig. 5:**
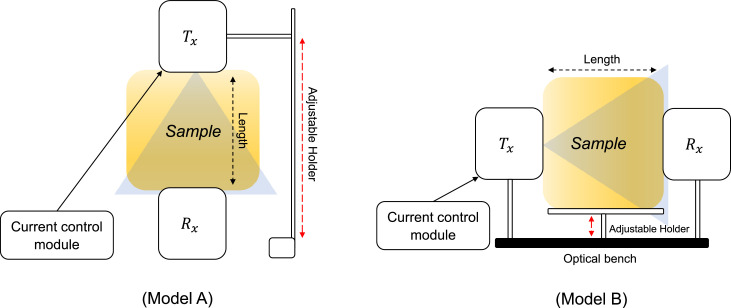
Block diagram of the test-bed, this dataset paper considers Model B.

There are two options for the test-bed placement, i.e., model A (Fig. 5a) or model B (Fig. 5b). Here, we consider model B as it allows flexibility to change the distance and position of $T_{x}$ or $R_{x}$ in horizontal axis direction. The block diagram of the test-bed used in this dataset paper is shown in Fig. 5(b); it consists of a $T_{x}$, $R_{x}$, current control (i.e., LED driver), and propagation medium (i.e., *ex-vivo* pork meat sample or phantom). The channels considered in this paper are free-space, optical phantom, and ex-vivo pork meat samples. The characterization data in the developed in-body OWC test-bed produces a dataset paper. As shown in Fig. 5(b), the $T_{x}$ and $R_{x}$ are positioned in a strict line-of-sight configuration, with the propagation medium placed in between or without them for a free-space measurement scenario. In the dataset, the free-space (without any tissue medium) is a baseline measurement of optical power to establish reference values.

#### 4.1.2 Calibration issues

In general, calibration of equipment for a test-bed is critically important. Uncalibrated equipment may lead to incorrect or invalid reading results (inaccuracy). The calibrated equipment ensures reliable and consistent data collection because errors in measurements are identified and corrected; it establishes traceability of measurements to standards, ensuring compliance with reproducibility. With regard to this, we carefully consider calibration issues to generate reliable and reproducible datasets.

This section is based on the discussion with technical support engineers from Thorlabs Europe in the email (i.e., *europe@thorlabs.com*) in March 2024. It is highly recommended that the optical power meter (PM100D) and optical sensor (S121C) be recalibrated every year. Calibration verifies that the equipment provides accurate measurements by comparing its output against a known standard of higher accuracy. In the calibration work, the original company compared the measurement results from our optical power meter to their trusted reference sensor to calibrate the sensor connected to the power meter.

The information on the valid date of calibration is labelled on the items or calibration certificate. It is also visible in the dedicated software if we connect the sensor to the console and the meter to a computer. The optical power meter software is free to download from Thorlabs’ website. We are also be able to display this on the PM100D by navigating to system settings and then selecting either “console info” or “sensor info”. The calibration date should be ensured before the start. If it is obsolete, the calibration can be ordered through the website by inputting important information, such as the part number and serial number, in the provided form, e.g., part numbers CAL-PD and CAL-PM1. After making a request, the Thorlabs administrator will reach out to coordinate the return of the item(s) for calibration. In our case, the calibration of the sensor S121 and the PM100D was completed in May 2024. Subsequently, the experiment was conducted in June 2024. Given this valid calibration timeline, the measurements obtained are considered reliable.

### 4.2 Step 2: preparation and safety concerns

Preparations encompass using various instruments to construct an in-body OWC test-bed, while safety concerns relate to technical safety in conducting experiments dealing with NIR exposure.

**Fig. 6:**
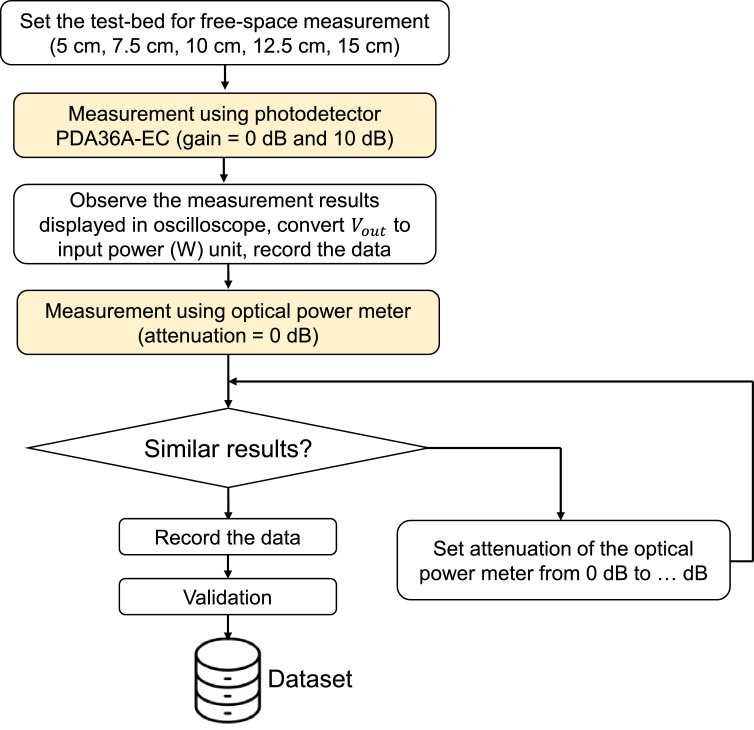
Methodology used in generating the dataset.

The $T_{x}$ side implemented an LED driver, i.e., DC2200, and a single beam-mounted NIR LED that has 810 nm of wavelength, i.e., M810L3. We employed two types of $R_{x}$ devices for comparison: a photodetector module and an optical sensor. The methodology used to generate the dataset is shown in Fig. 6. We conducted measurements several times until getting precise results. After the test-bed is ready, before starting, it is encouraged to ensure that all electrical equipment is grounded properly and avoid overloading circuits connected to the test-bed.

We used a sensor S121C having Ø9.5 mm [28] and connected to an optical power meter console, i.e., PM100D; it is a portable energy and optical power meter specifically designed to measure the optical power emitted by laser light or other light sources that exhibit monochromatic or near-monochromatic characteristics. The second $R_{x}$ device is a photodetector, i.e., PDA36A-EC, and it is connected to an oscilloscope. It is a switchable gain photodetector; it contains a trans-impedance amplifier with low noise, which is integrated within the photodetector module.

The DC2200 can be controlled using the front panel and a digital display. The power level of LEDs was considered safe for an in-body OWC system as it was below the maximum allowable limit according to the ANSI.Z136.1-2007 standard [19,31]. The incident power of the LED varied depending on the electrical current. The NIR LED was driven by the DC2200. We used constant current mode with a maximum of 500 mA. Theoretically, the LED will generate 375 mW (3.75W) of incident optical power if it is driven at 100% of current, i.e., 500 mA. Then, varying the current level into 90%, 80%, 70%, 60%, 50%, 40%, 30%, 20%, and 10% will result in 337.5, 300, 262.5, 225, 187.5, 150, 112.5, 75, and 37.5 mW, respectively. We then measured the incident power to determine the actual value using sensor S121 integrated with PM100D. The measurement results are presented in the file “Raw data - optical test-bed in-body communications (free-space)” sheet 1.

During the experiment, we strictly adhered to protective eyeglasses standards (certified laser safety glasses provided by Thorlabs) to minimize the potential risk of eye injuries from exposure to high radiation levels [32,33], i.e., Thorlabs LG9 certified ANSI.Z136 laser safety glasses [34]. Moreover, it is advised that *ex-vivo* pork meat samples and phantom be handled using proper gloves and that hygiene standards be maintained to avoid contamination. The experiments were conducted in a dark condition, which is approximately 0 lux.

As shown in Fig. 6, we first conducted measurements in free-space to establish a baseline, using a photodetector module connected to an oscilloscope at precise distances of 5, 7.5, 10, 12.5, and 15 cm between the NIR LED and the photodetector module. The gain setting of the photodetector module was set to 0 dB and subsequently adjusted to 10 dB. The NIR LED was operated at a predetermined current, i.e., 50 to 500 mA with increments of 50 mA. The output signal from the photodetector module, as displayed on the oscilloscope, was recorded. We then repeated 10 times at each 1) distance, 2) gain setting, and 3) current setting for NIR LED to manage the erroneous measurement. After calculating the average signal, the photodetector module was replaced with an optical sensor connected to an optical power meter. Initial measurements were conducted at attenuation = 0 dB.

Subsequently, additional experiments were performed by varying the attenuation until the readings aligned with those obtained from the photodetector module, resulting in an attenuation value of –4.30 dB. The optical power measurements at attenuation levels of –4.30 dB and 0 dB were compiled into a dataset. The data validation process involved a comparative analysis between the repeated measurements obtained from the photodetector and the optical power meter. Only the most accurate data were recorded, ensuring that the best fit for the final dataset. Similar measurement procedures were employed for assessments involving phantoms and *ex-vivo* pork meat samples.

### 4.3 Setup for a photodetector

Measuring an optical power using a PDA36A-EC requires careful consideration as we can not directly obtain an optical power unit instead of a $V_{out}$ unit, and needs to extract it. Therefore, we can manage the reading capability by adjusting its amplifier gain. The mentioned photodetector module provides amplifier gain in increments of 10 dB, ranging from 0 – 70 dB, available from the knob. It is essential to note that adjusting the gain setting impacts its bandwidth, as indicated in Table 3. Specifically, increasing the gain leads to narrowing the bandwidth, which renders certain gain settings unsuitable for use. Consequently, the usable gain settings in this test-bed are limited to 0 dB or 10 dB. The PDA36A-EC has an impedance (Z) of 50 Ω (denoted as $R_{source}$), which requires caution when connecting it to a particular load as it determines its input optical power value. The photodetector's gain values are 750 V/A and 2380 V/A (with each having ±2%) when the knob is set to 0 dB and 10 dB, respectively, with the load being the same value, which is 50 Ω. The purpose of the photodetector module is to detect optical signals emitted by an 810 nm NIR LED. The typical peak responsivity and the responsivity of the photodiode used in the PDA36A-EC are 970 nm and 0.65 A/W, respectively. However, based on the datasheet [24], the responsivity at 810 nm is selected at approximately 0.5 A/W (close to 800 nm).

**Table 3:** Information on the photodetector module.

Parameters	0 dB setting	10 dB setting
Gain value (50 Ω of *Z*)	0.75 × 10^3^ V/A ±2%	2.38 × 10^3^ V/A ±2%
Photodiode’s responsivity in 810 nm	0.5 A/W	
Bandwidth	10 MHz	5.5 MHz

The generated photocurrent is converted into a $V_{out}$ by a load resistance, then it is visualized and read on the oscilloscope. The choice of the photodetector and load resistance determines the speed at which the system responds. The oscilloscope used in the experiment provides two options for input impedance: 50 Ω and 1 MΩ, which can be selected through the provided button [30]. For this dataset paper, the load impedance of 50 Ω was selected and referred to as $R_{load}$ (Table 4) while $R_{source}$ is referred to as impedance output from the photodetector, i.e., 50 Ω either (Table 4). The maximum $V_{out}$ of the photodetector module is 10V; hence, if the light source is too close to the photodetector’s sensitive area, the $V_{out}$ will saturate. For this reason, we did not measure the incident optical power from NIR LEDs using a photodetector in the dataset.

**Table 4:** Information on the impedance of the photodetector and oscilloscope.

Parameter	Value	Description
$R_{load}$	50 Ω	Z input of the oscilloscope
$R_{source}$	50 Ω	Z output of the photodetector

The gain scale factor is determined by the values of $R_{source}$ and $R_{load}\;$as expressed in (1). The input power of the photodetector is formulated using (2), where $V_{out}$ is the output voltage from the photodetector module read via an oscilloscope. The gain scale factor is the value obtained from (1). Gain is a reference value, which refers to Table 3, and the responsivity of the photodiode is dependent on the wavelength of the LED used (810 nm in this case, with a responsivity of about 0.5 A/W).

(1) $$\text{Gain}\;\text{scale}\;\text{factor}\;=\frac{R_{load}}{R_{load}+R_{source}},$$

(2) $$\text{Input}\;\text{Power}\;\left(\text{Watt}\right)=\frac{V_{out}}{\text{Photodiode's}\;\text{responsivity}\times \text{Gain}\;\text{value}\times \text{Gain}\;\text{Scale}\;\text{factor}}$$

### 4.4 Setup for an optical sensor

Measuring optical power using the PM100D is more flexible than a PDA36A-EC since it is originally designated to read the optical power. The sensor S121 is then connected to the mentioned optical power meter. We set the gain factor of the PM100D to two values for comparison, i.e., 0 dB and –4.30 dB. The input aperture was set to Ø9500 nm to match the sensor’s specification. Furthermore, we set the bandwidth to high-bandwidth mode (BW Hi). The mentioned optical power meter allows the user to input an attenuation or gain factor within a range of 60 to –60 dB. The beam diameter has the capability to be adjusted within a range of 10 µm to the diameter of the sensor aperture in the provided port. This feature enables the visualization of the laser or probe power in its original form without requiring a filter or beam splitter in the system. Additionally, it can be utilized to expand the measurement range of a power or energy sensor by incorporating a calibrated filter [29].

### 4.5 Free-space measurement setup

For a data-based measurement on a free-space setting, we used the mentioned optical power meter to measure three parameters: optical power meter (in Watts and dBm) and received power density (W/cm^2^). Then, the measurement results of optical power meter readings were compared to the photodetector; however, the photodetector module can solely measure the optical power in Watt units using (2). Measurement in free-space channel was conducted at 5, 7.5, 10, 12.5, and 15 cm. On the other hand, incident optical power was measured at 0.1 cm using an optical power meter.

### 4.6 Measurement setup using samples (phantom and pork meat)

**Fig. 7:**
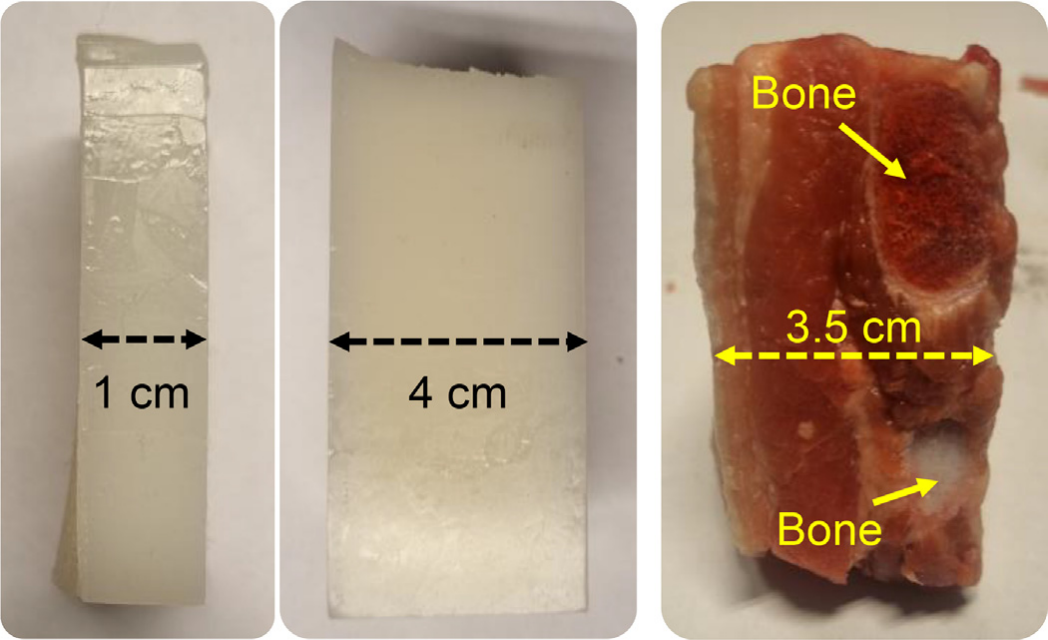
Phantom and *ex-vivo* pork meat samples.

Besides the free-space measurement, we used a tissue-mimicking phantom (4 cm and 1 cm thicknesses) and *ex-vivo* pork meat samples presented in the dataset, as shown in Fig. 7. A phantom used in this study mimicked human soft tissue; it was originally introduced in [1]. This phantom is created from a polyvinyl chloride plastisol (PVCP) mixture. It demonstrates optical absorption and scattering properties across the 400 – 1100 nm wavelength, aligning with the typical characteristics found in the human soft tissue [2]. The pork meat samples are fresh samples purchased from the local market at an initial temperature of approximately 7.9°C (measured using Klein Tools MM400).

### 4.7 Step 3: measurements

**Fig. 8:**
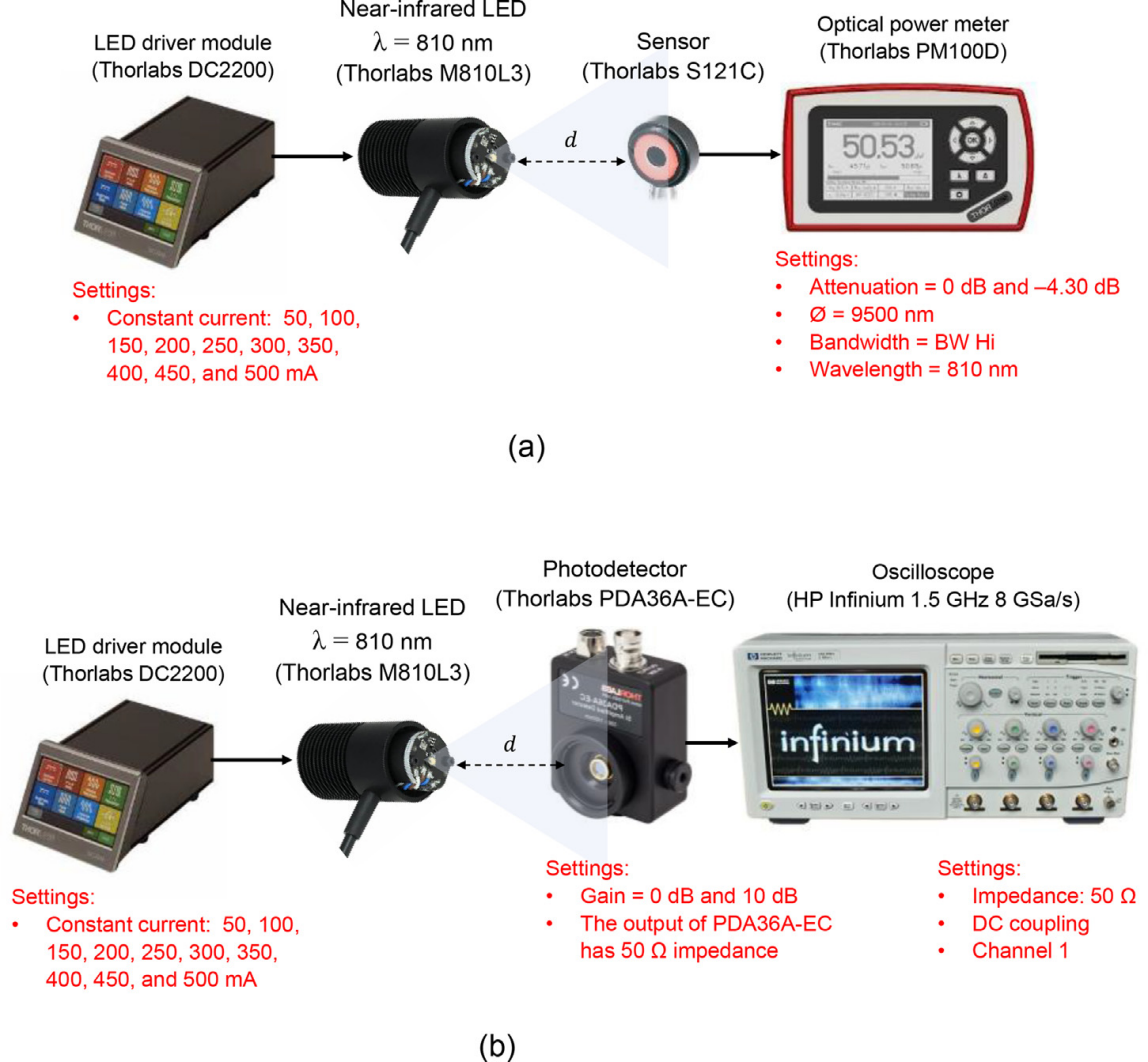
Experimental setup: (a) measurement using an optical power meter; (b) measurement using a photodetector module.

The next step is the measurement campaign without (free-space settings) and with the sample channels after the setups are ready. The equipment used for data collection, as presented in this dataset paper, including the photodetector module, oscilloscope, and optical power meter, provides insights into the measurement process. The experimental test-bed comprised an $T_{x}$ and $R_{x}$ devices. The PDA36A-EC was connected to the oscilloscope’s channel using a BNC cable (via *CH_1_*) in order to obtain measurement data of $V_{out}$ (Fig. 8a). Conversely, the PM100D allows for direct visualization of the data in its display (Fig. 8b). The equipment was placed on an optical bench (Thorlabs, MB3060/M). As mentioned before, in a free-space setting, the optical distance was changed to 0.1 cm for measuring the incident power and then subsequently varied to 5, 7.5, 10, 12.5, and 15 cm.

**Fig. 9:**
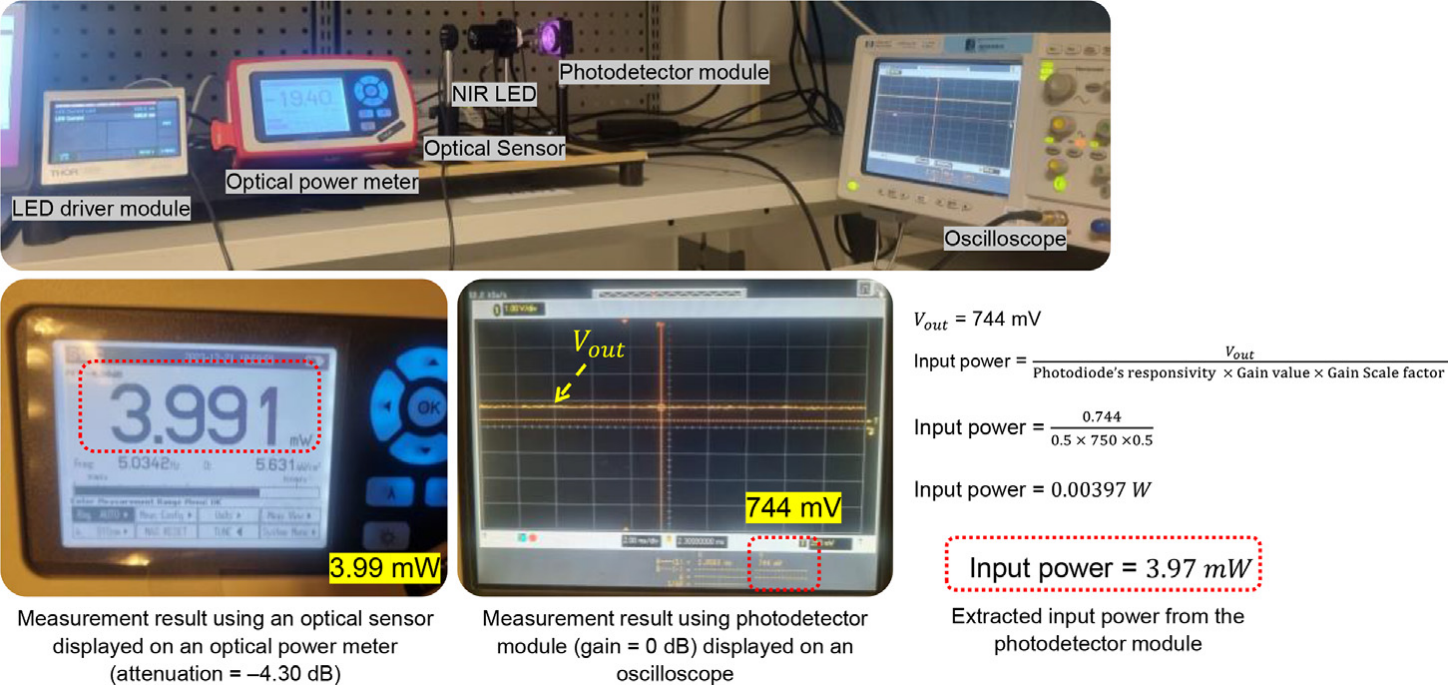
Reading measurement methodology.

The measurements on a free-space were done separately. First, we considered a photodetector module as a $R_{x}$ unit, with the knobs adjusted to 0 dB and 10 dB. The input current for the LED was controlled by adjusting the LED driver module, e.g., 50, 100, 150, 200, 250, 300, 350, 400, 450, and 500 mA. The $V_{out}$ was measured by the oscilloscope and subsequently converted to input optical power using (1). Afterward, the graphs were plotted, showing the function of optical power measured by a photodetector module against the LED’s current. Fig. 9 depicts the reading measurement methodology for measurement using a photodetector module; it shows an example of how the dataset was generated in this study.

Second, the photodetector module is replaced with the optical sensor with the precise location. The optical power meter reading was obtained using the sensor S121C, and the results (optical power in mW, dBm, and power density in mW/cm^2^) were directly read from the PM100D.

**Fig. 10:**
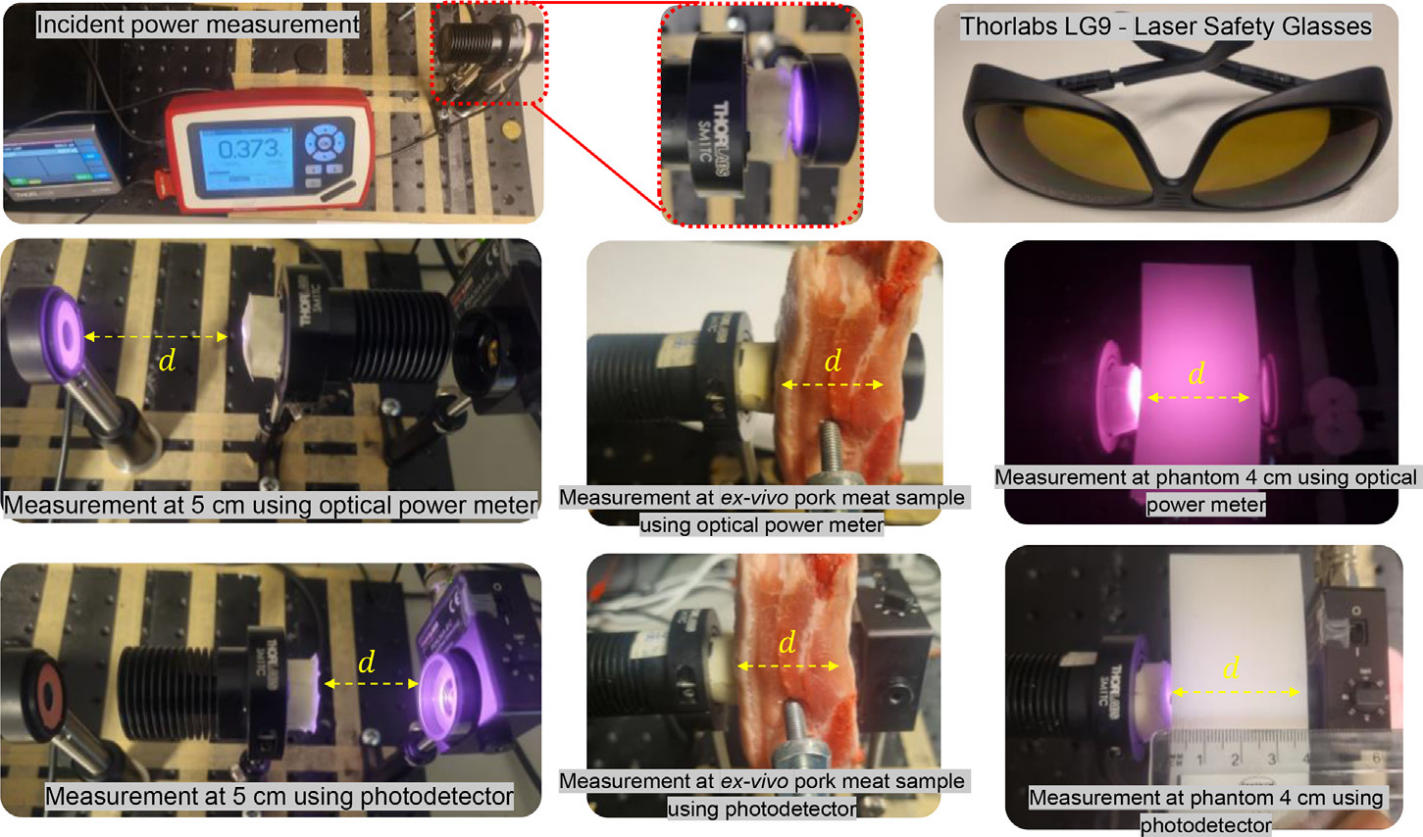
Photographs of experimental work in the laboratory.

As shown in Fig. 9, the optical power measurement reading on the PM100D display indicates 3,991 mW. Concurrently, the $V_{out}$ of 744 mV from the PDA36A-EC is observable on the oscilloscope display, which is subsequently converted to an input power unit using (2), resulting in 3.97 mW. The results are quite similar. The same procedure was applied to the measurements using the samples (i.e., phantom and *ex-vivo* pork meat). Fig. 10 shows the experimental setup in the laboratory.

For the photodetector module to serve as a measuring instrument, the data obtained must be multiplied by a factor of approximately 2.7. On the other hand, it can also be summarized that the optical power reading by the PM100D with attenuation = –4.30 dB is equal to the reading of PDA36A-EC in any gain (here, we consider 0 dB and 10 dB). Alternatively, this can be expressed in (3) or (4). The data obtained demonstrates that (3) and (4) are applicable when utilizing samples to demonstrate in-body OWC systems given on the provided test-bed, where the power reading obtained from the optical power meter through phantom and biological tissue is equivalent to 2.7× that of the photodetector module.

(3) $$\begin{array}{ccc} Optical\;power\;meter\;measurement\;\left(attenuation\;0\;dB\right) & = & 2.7\;\times Optical\;power\;meter\;of\\ & & photodetector\;\left(0\;dB\;or\;10\;dB\right), \end{array}$$

Or it can be expressed as:

(4) $$\begin{array}{ccc} Optical\;power\;meter\;measurement\;\left(attenuation-4.30\;dB\right) & = & Optical\;power\;meter\;of\\ & & photodetector\\ & & \left(0\;dB\;or\;10\;dB\right). \end{array}$$

### 4.8 Step 4: generated datasets

The dataset is then generated through the measurement campaigns referred to Fig. 6. This includes dataset experimental in-body OWC test-bed for research purposes on:

• Free-space channel (incident optical power, measurements on 5, 7.5, 10, 12.5, and 15 cm),
• phantom (1 cm and 4 cm thicknesses) and
• *ex-vivo* pork meat channels.

The dataset available in the repository is presented in Excel as described in detail above. However, the data in this paper are presented on a graph showing the difference between the photodetector module (set to 0 dB and 10 dB of gain) and the optical sensor (set to –4.30 dB and 0 dB of attenuation) reading results. The description of the raw dataset in Excel files is elaborated in the section data description.

### 4.9 Free-space

**Fig. 11:**
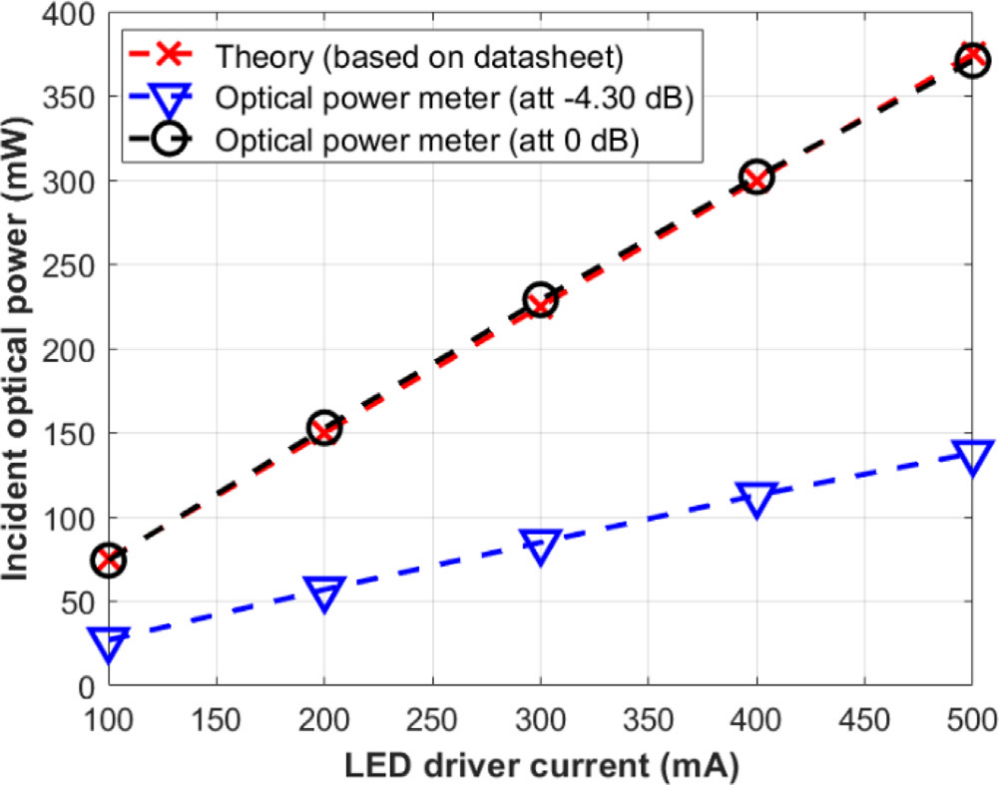
Measurement results of LED characteristics in mW unit.

Fig. 11 shows the characteristics of LED used in this dataset paper. The measurement results are very close to the theory when it is adjusted to an attenuation = 0 dB. The impact of the attenuation setting within the PM100D on the measurement results should be acknowledged. To ensure accuracy and maintain consistency with the original value, it is recommended to keep the PM100D adjustment to attenuation = 0 dB, as this setting allows for a precise reading of incident power with the dataset. In this sense, measuring incident power should be done using an attenuation = 0 dB.

**Fig. 12:**
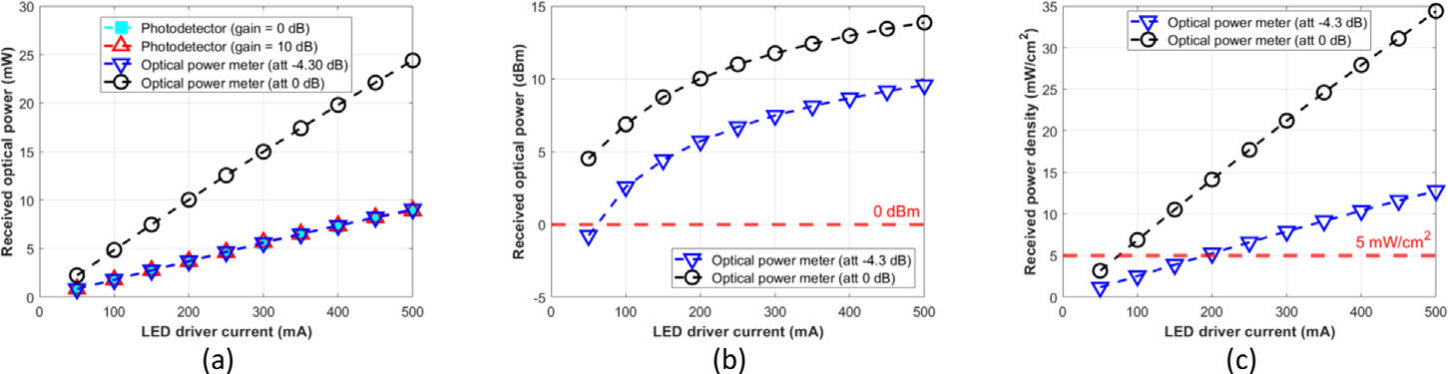
Measurement results at free-space 5 cm: (a) mW unit; (b) dBm unit; (c) mW/cm^2^ unit.

**Fig. 13:**
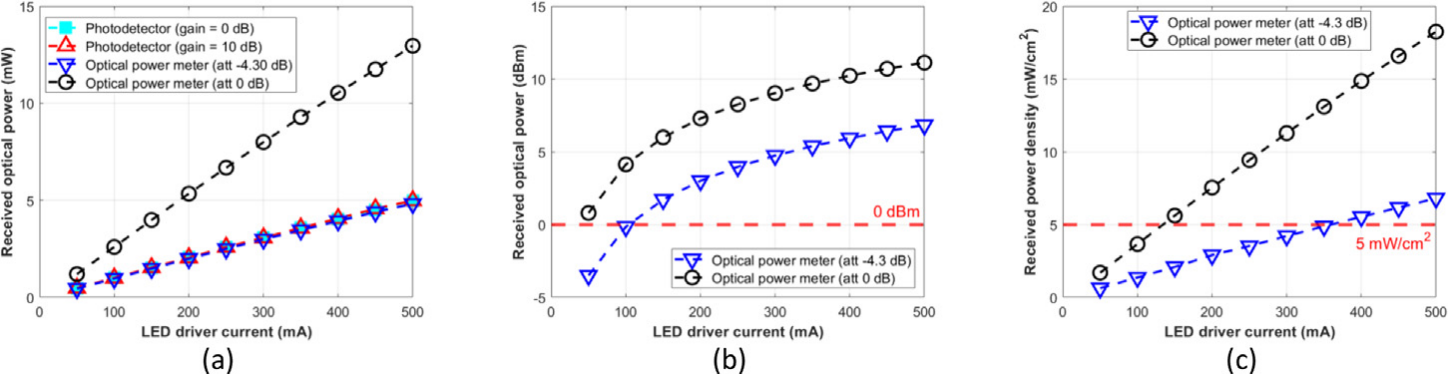
Measurement results at free-space 7.5 cm: (a) mW unit; (b) dBm unit; (c) mW/cm^2^ unit.

**Fig. 14:**
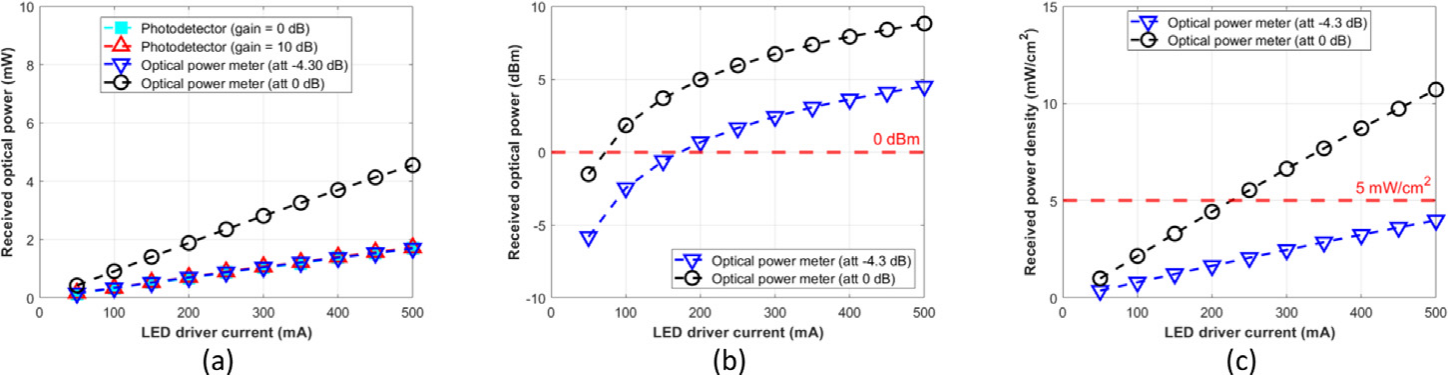
Measurement results at free-space 10 cm: (a) mW unit; (b) dBm unit; (c) mW/cm^2^ unit.

**Fig. 15:**
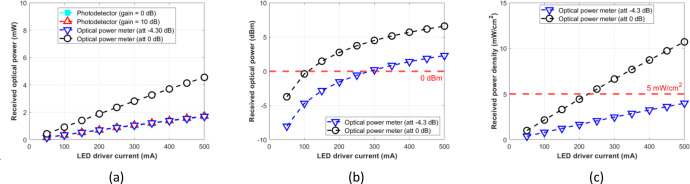
Measurement results at free-space 12.5 cm: (a) mW unit; (b) dBm unit; (c) mW/cm^2^ unit.

**Fig. 16:**
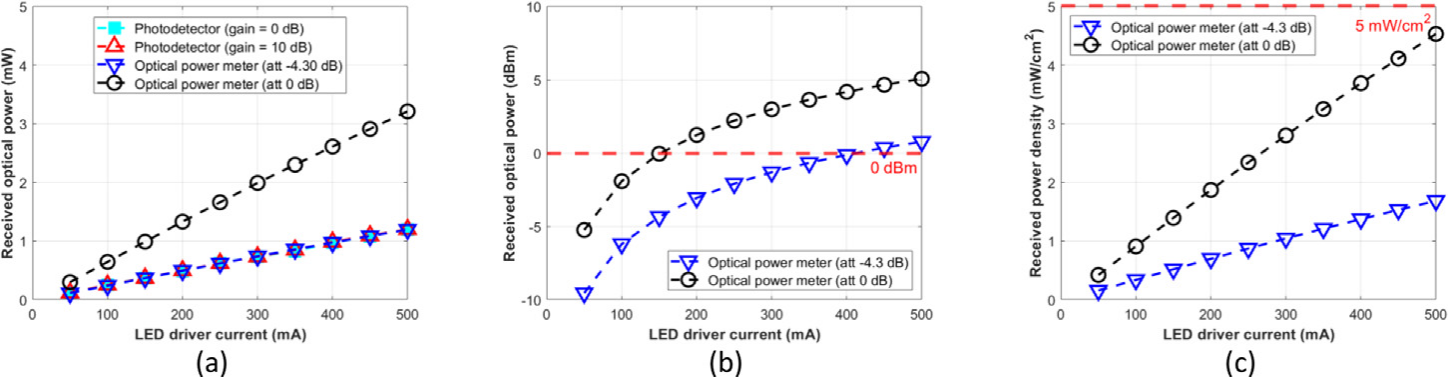
Measurement results at free-space 15 cm: (a) mW unit; (b) dBm unit; (c) mW/cm^2^ unit.

The attenuation setting to –4.30 dB of the optical power meter resulted in a similar result to the photodetector module with gain = 0 dB or 10 dB at the optical distances of 5, 7.5, 10, 12.5, and 15 cm. Therefore, for the photodetector module to function as an optical power meter, its reading result should be normalized as formulated by (3), which is multiplied by the factor 2.7. Further, we expect that the normalized optical power can be used as a reference for various experimental setups, such as mapping the incident power on the LED driver for OWPT research through the biological tissue without phantoms or pork meat samples. Figs. 12, 13, 14, 15, and 16 show the measurement results at 5, 7.5, 10, 12.5, and 15 cm, respectively.

### 4.10 Optical phantoms

**Fig. 17:**
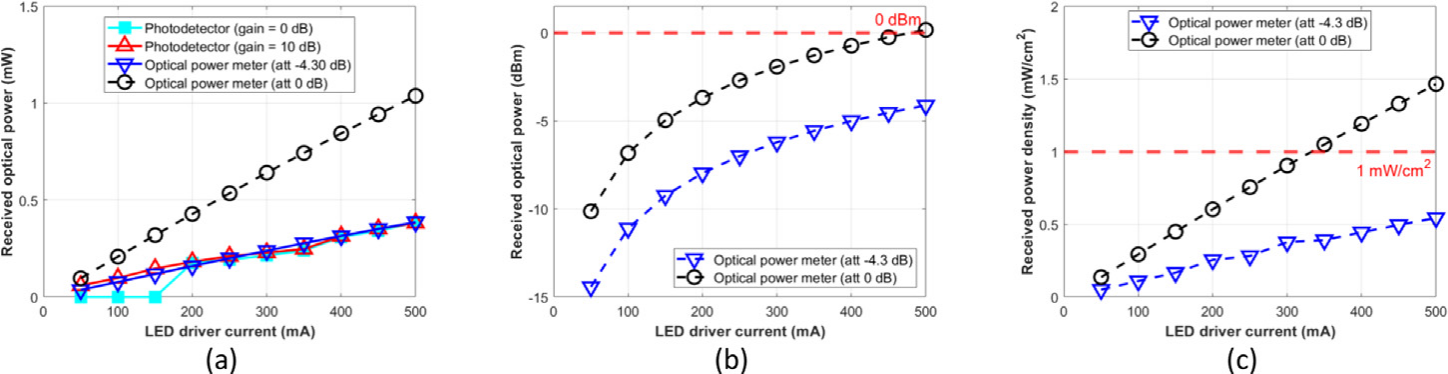
Measurement results on 4 cm phantom thickness: (a) mW unit; (b) dBm unit; (c) mW/cm^2^ unit.

In the context of data-based measurements using phantom, the procedure is implemented in the same way as in the free-space scenario. The optical power is measured using two different $R_{x}$ devices (i.e., optical power meter and photodetector module) in a separate measurement. Still, the channel distance was set to fixed, which is 1 cm and 4 cm, depending on the phantom thicknesses. The phantom sample’s surface was illuminated directly by NIR LED, while the $R_{x}$ device was positioned on the opposing side. Figs 17(a), (b), and (c) show the measurement results on the 4 cm thickness phantom in Watt, dBm, and mW/cm^2^ units, respectively.

**Fig. 18:**
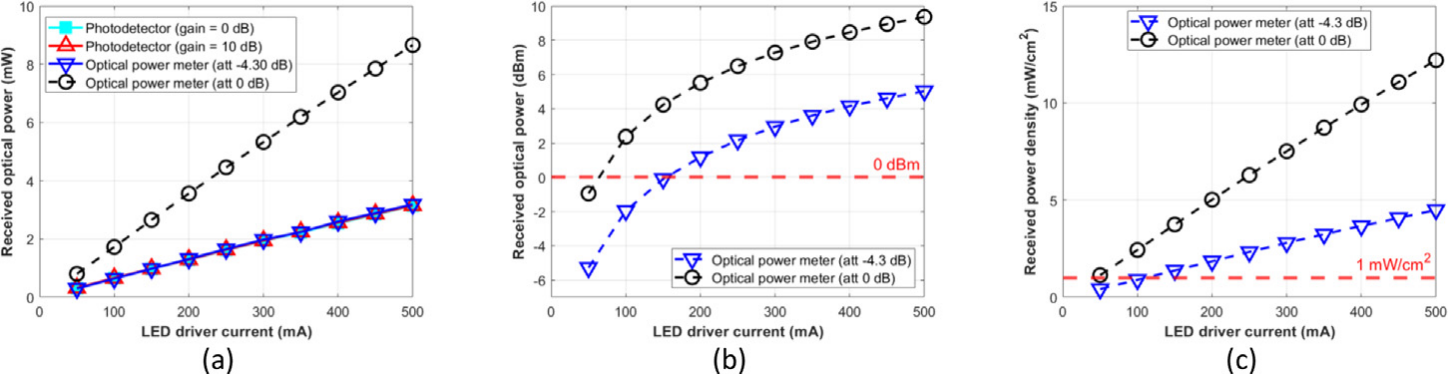
Measurement results on 1 cm phantom thickness: (a) mW unit; (b) dBm unit; (c) mW/cm^2^ unit.

Figs 18(a), (b) and (c) show the measurement results on the 1 cm thickness phantom in Watt, dBm, and mW/cm^2^ units, respectively.

### 4.11 Biological tissue

**Fig. 19:**
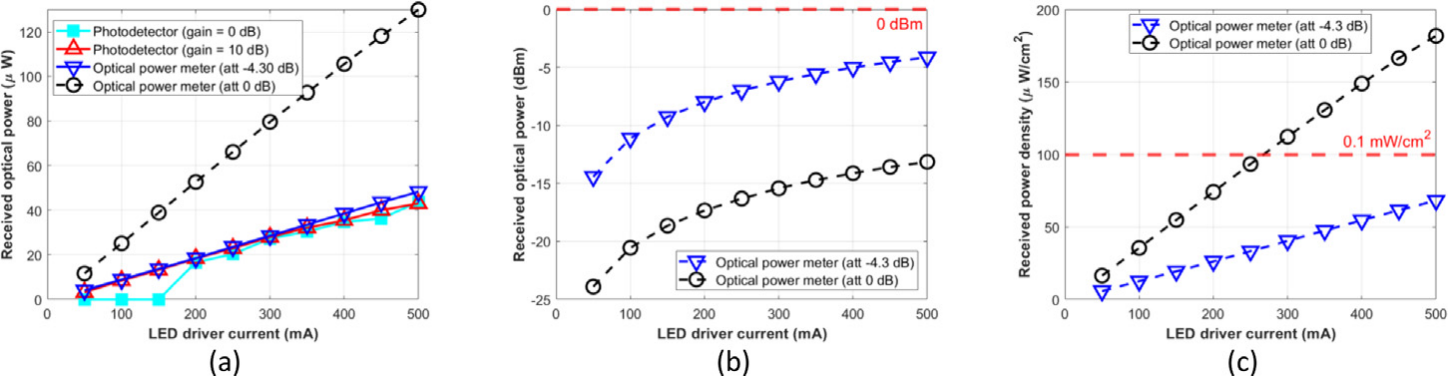
Measurement results on *ex-vivo* pork meat sample: (a) µW unit; (b) dBm unit; (c) µW/cm^2^ unit.

**Fig. 20:**
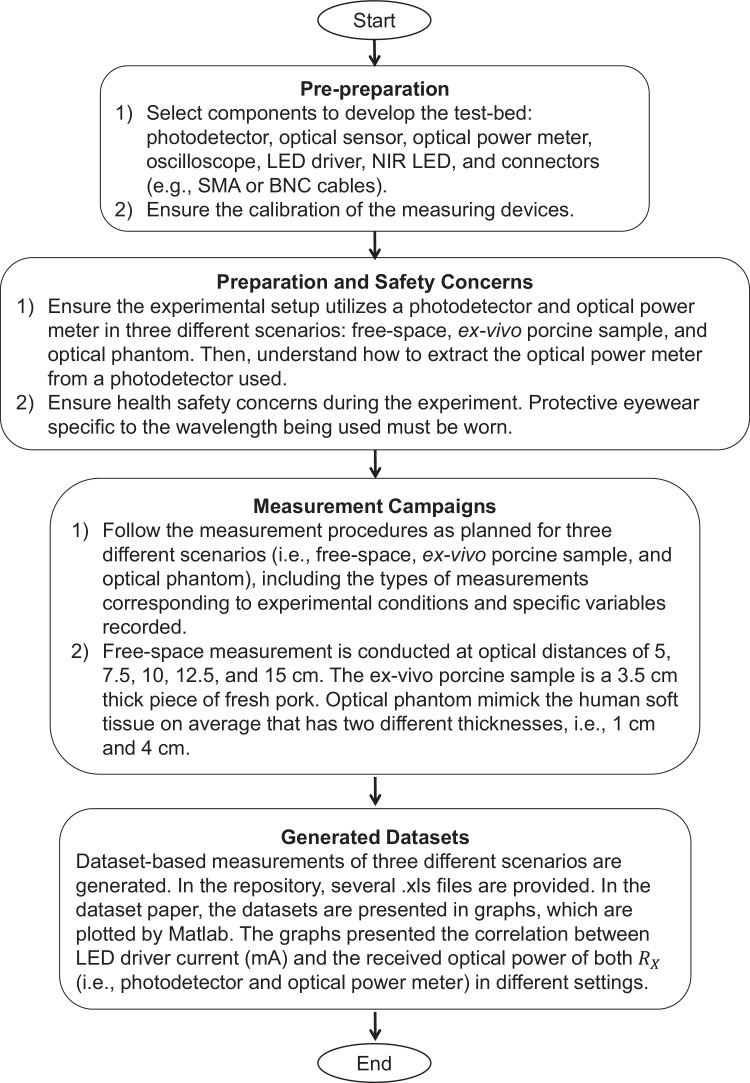
A flowchart visually described the design, material, and methods used in this dataset paper.

The procedure for measuring biological tissue is implemented in the same way as in the phantom scenario. Figs 19(a), (b), and (c) show the measurement results on *ex-vivo* pork meat samples in Watt, dBm, and µW/cm^2^ units, respectively.

## Limitations

In this dataset paper, we have described in detail the protocol and methodology for generating datasets related to the test-bed preparation for in-body OWC research. The experimental setup includes details on the test-bed demonstrator components, such as the $T_{x}$ and $R_{x}$ front-ends supported by Thorlabs. The LED current control and the photodetector gain settings are specified in this dataset. The measurement scenarios were conducted in free-space channels, phantom, and biological tissues using *ex-vivo* pork meat samples. The data are accessible through the Mendeley repository with the provided data identification number and direct URL. In contrast, all raw data related to the tables, graphs, images, and charts is provided in the manuscript.

However, there are limitations in the dataset. First, the responsivity of the optical sensor (i.e., S121) used in this study is wavelength-dependent, meaning that accurate optical power measurements require the user to input the correct wavelength reading into the power meter console; in this case, we set 810 nm in the optical power meter. The photodetector module (i.e., PDA36A-EC) used in this study has a peak wavelength that is different from that of an optical sensor. In principle, the mentioned photodetector module converts incoming incident optical power from NIR LED into a measurable electrical current. This current is then processed by an internal amplifier, which outputs a voltage proportional to the input current. Then, the received optical power is derived from the $V_{out}.\;$The PDA36A-EC directly measures the received optical power without requiring a peak wavelength setting. Therefore, the conversion results will likely differ when researchers use different photodetector module types.

Second, using the mentioned photodetector module to measure received optical power requires extremely careful placement, as the $V_{out}$ reading is sensitive to misalignment. We experienced that any slight misalignment causes a difference in reading accuracy. Even in some measurement settings, as presented in “Raw data – Optical test-bed In-body communications (Phantom) file”, the $V_{out}$ is zero, meaning that the PDA36A-EC accuracy is low. The possible reason is that, essentially, PDA36A-EC is primarily used for applications that require the detection of light intensity rather than precise optical power measurements. Its accuracy can be limited by factors such as signal amplitude and noise. It was described in the datasheet that the gain value has a ±2% tolerance [24], in this dataset, we did not consider this tolerance in the calculation. Besides, the photodiode’s responsivity is one of the key parameters applied in (1); a responsivity value of 0.5 A/W is typically specified at 800 nm based on the response curve provided in the datasheet [24]. We adopted this value as a reference despite our experiment using a wavelength of 810 nm. The slight difference in wavelength may contribute to the difference in the measurement results. The mentioned photodetector is more suited for relative measurements rather than absolute power accuracy, while a PM100D is dedicated to calibration routines; thus, the accuracy is very high. According to the Thorlabs technical support team, the PDA36A-EC is not calibrated by the National Institute of Standards and Technology (NIST), as compared to sensor S121. Considering the factors outlined earlier, we assume that the accuracy of the PDA36A-EC will not be within ±3%, as is typical for most power meters provided by Thorlabs. Users are advised to recharacterize their test-bed if they employ different photodetector modules, NIR LED, and its use cases using the methodology proposed in this dataset paper, as shown in Fig. 6.

To ensure consistency in the precision of measurement results. A new testbed to support this requirement might be essential to consider in future work. The testbed may be developed by a 3D-printed enclosure (like a box) that houses a tissue sample. The transmitter part (i.e., NIR LED) is mounted at the top of the enclosure and pressed directly against the tissue sample. In contrast, the receiver part (photodetector or sensor) is fixed in position at the bottom. This vertical arrangement ensures reliable and repeatable alignment of the transmitter and receiver components, significantly reducing setup time and alignment errors compared to the horizontally arranged proposed method.

Third, the PDA36A-EC features a ring around the photodetector area (SM1T1 Coupler with SM1RR retainer ring), allowing for integration with Thorlabs’ optomechanical components, such as lenses. This ring creates an air gap of approximately 7.1 mm, preventing direct contact between the photodetector surface and *ex-vivo* samples or optical phantoms during tests. To ensure comparable results, the sensor S121 was positioned about 7 mm from the sample’s backside. The PDA36A-EC has been discontinued and replaced by the PDA36A2 (https://www.thorlabs.com/thorproduct.cfm?partnumber=PDA36A2), which retains the same adjustable gain capability, photodiode specifications, and performance but features an updated housing. Notably, the PDA36A2 allows the removal of the front mounting ring, enabling direct contact between the detector and samples for similar measurements without an air gap.

Fourth, the forthcoming dataset should incorporate statistical measures, including standard deviation, to ascertain the accuracy of optical power readings for both $R_{x}$ devices. Fourth, it is essential to emphasize that the heating of *ex-vivo* pork sample meat should achieve a temperature of approximately 37°C, aligning with the average human body temperature [35]. In this dataset, fresh pork meat obtained from the local butcher was measured at approximately 7.9°C and gradually warmed to room temperature during measurements, reaching approximately 14.7°C.

Although this study has certain limitations, the results provide valuable and commendable contributions, specifically to the measurement methodology in OWC studies.

## Ethics Statement

The authors declare that there are no ethical issues regarding this dataset. The authors confirm that their research did not involve human or animal life subjects. We used a phantom, which is an object that mimics the human body. On the other hand, the fresh pork meats used in this study were obtained from a local market that sells various types of meat, including pork meat. For this reason, it is not classified as an animal experiment. Any mention of specific commercial products, manufacturers, trademarks, or other entities in this dataset paper should not be interpreted as an endorsement, recommendation, or preference by the research units at the University of Oulu, Finland. Furthermore, the views and opinions expressed by the authors are their own and do not necessarily reflect the official stance or perspectives of the University of Oulu.

## CRediT Author Statement

**Syifaul Fuada:** Conceptualization, Methodology, Validation, Formal Analysis, Data curation, Investigation, Writing – original draft; **Mariella Särestöniemi:** Supervision, Resources, Writing – review & editing; **Marcos Katz:** Writing – review & editing, Supervision, Funding Acquisition.

## Data Availability

Mendeley DataTest-bed Dataset for Optical-Based In-Body Communications Research (Original data).

## Appendix

We have described the experimental design, materials, and methods in textual form. This appendix provides a diagram illustrating the test-bed configuration and a step-by-step measurement process, which enhance comprehension (shown in Fig. 20), particularly for researchers new to this field.
